# Biomechanical Differences Among Collegiate Sprinters Developed Through Specialized and Diversified Training Pathways

**DOI:** 10.3390/s26061906

**Published:** 2026-03-18

**Authors:** Huashuai Li, Shaoze Zheng, Shihao Wang, Qingyang Cao, Ruiyang Zhang

**Affiliations:** College of Physical Education and Sports, Beijing Normal University, Beijing 100875, China; 202321070032@mail.bnu.edu.cn (S.W.); 202522070002@mail.bnu.edu.cn (Q.C.); 202411070007@mail.bnu.edu.cn (R.Z.)

**Keywords:** biomechanics, reactive strength, training specialization, isokinetic strength, stretch–shortening cycle, postural control

## Abstract

**Highlights:**

**What are the main findings?**

- Sprinters following a specialized athletic development pathway exhibited faster force production timing (shorter time-to-peak torque) in the nondominant knee extensors, while peak torque was comparable to peers following a diversified physical education-centered pathway.
- Sprinters following a specialized athletic development pathway demonstrated superior stretch–shortening cycle performance (higher reactive strength index, shorter ground contact time, greater leg stiffness) together with phase-specific electromyography differences during drop jumps.

**What are the implications of the main findings?**

- The sprint-performance advantage associated with specialized training appears to be linked more to neuromuscular reactivity/coordination and stretch–shortening cycle efficiency than to leg maximal strength (as indexed by isokinetic peak torque at 60°/s), suggesting that training should prioritize rapid force generation and efficient elastic energy utilization.
- For participants following a diversified physical education-centered pathway, training should emphasize reactive strength development (plyometrics with short ground contact time) and sensorimotor/postural-control work to enhance rapid force transfer and narrow performance gaps.

**Abstract:**

This study compared collegiate sprinters from two common admission routes in China to identify pathway-associated differences that may inform subsequent training for athletes entering via the Physical Education College Entrance Examination pathway. Twenty male collegiate sprinters were allocated to a Sports Independent Enrollment group and a Physical Education College Entrance Examination group, with ten participants in each. Participants completed isokinetic knee testing, drop jump tests, static balance tests, and drop jump electromyography assessment. Isokinetic outcomes were largely comparable between groups, although the Sports Independent Enrollment group showed faster time to reach peak torque in the nondominant-side knee extensors. In drop jumps, the Sports Independent Enrollment group demonstrated higher reactive strength, shorter ground contact time, greater leg stiffness normalized to body weight, and shorter propulsion duration. Electromyography patterns differed between groups across movement phases. Balance differences were mainly observed under the single-leg eyes-closed condition in unadjusted comparisons, but none remained significant after false discovery rate adjustment. Overall, between-group differences were more evident in rapid force production and neuromuscular control than in the magnitude of isokinetic strength. These findings provide practical targets for designing subsequent training priorities for athletes entering through the Physical Education College Entrance Examination pathway.

## 1. Introduction

The Physical Education College Entrance Examination (PECEE) and Sports Independent Enrolment (SIE) are two special admission policies in China for student-athletes. Both admission routes are designed to integrate sports education with academic education, allowing student-athletes to leverage their athletic advantages to enter higher education.

Specifically, the SIE emphasizes the enhancement of sports specialization and competitive performance: this pathway is intended for candidates with a relatively specialized training background in a single registered sport, and therefore their pre-university preparation typically emphasizes long-term sport-specific practice, including technical skill acquisition and high-intensity conditioning aligned with competitive demands (such as plyometric exposure, repeated high-intensity efforts, and sport-specific movement patterns). In contrast, the PECEE places more emphasis on the comprehensive development of physical fitness: it refers to the mainstream provincial unified P.E. aptitude test used within the general college entrance system for applicants to PE-related majors. Although specific implementation details vary by province, a common “four-event” structure is adopted in many regions, comprising standardized, fitness-oriented items such as a 100 m sprint, standing triple jump or standing jump, standing shot put, and an 800 m run, with each item contributing a fixed proportion to the total test score in certain provinces. Accordingly, pre-university training for PECEE candidates typically follows a more generalist test-preparation profile, prioritizing performance in sprinting, jumping, throwing, and middle-distance running through repeated practice of these test items and broad physical conditioning (speed, explosive power, aerobic, anaerobic endurance), with comparatively less emphasis on sport-specific technical specialization than the single-sport recruitment route. The two educational pathways represent specialized development and comprehensive development in training models, respectively. Owing to the insufficient depth in the development of certain physical qualities within the comprehensive training model, compared with SIE students, PECEE students may exhibit differences in fundamental physical fitness and subtle aspects of movement execution. These differences can, to some extent, influence and even determine the future development direction and training methods of students.

Training models, load–injury relationships, and factors regulating athlete tolerance and development frameworks are critical to athletic success [1,2,3]. The ongoing academic discussion about specialized versus diversified training remains a focal point: In the athlete-development literature, the central debate is whether early single-sport specialization is necessary or advantageous, and the effect of early diversification on long-term performance and athlete health. Previous research indicates that early specialization before puberty does not significantly improve physical fitness in youth basketball players, and the timing of specialization onset is not significantly associated with subsequent fitness development [4]. Some elite figure-skating coaches argue that, rather than specializing too early, youth athletes should be encouraged to engage in broader early participation and diversified training; they cite concerns about the potential risks of early specialization and emphasize that long-term success depends more on trainable capabilities and development opportunities shaped by cultural context [5].

While early specialization may have some negative effects on an athlete’s development, specialized training is still crucial for competitive success. Previous studies have shown that an athlete’s training model largely affects their physical and skill development. For instance, middle- and long-distance runners typically excel in terms of aerobic capacity, whereas basketball and weightlifting athletes often dominate in terms of anaerobic strength [6]. Even during throwing events, there are notable differences: shot putters have excellent upper body strength but weak speed, discus throwers benefit from height but may lack core strength, javelin throwers have explosive power but a low BMI, and hammer throwers are stable in terms of core strength but have a weaker jumping ability [7]. Within the same discipline, differences in physical and skill performance can also occur because of variations in training objectives or levels. Elite freestyle skiing aerialists, for example, demonstrate significantly superior body composition, anaerobic metabolism, lower limb explosiveness, core strength, and agility than regular athletes do [8], and elite athletes also perform better in terms of reaction inhibition, indicating a greater capacity to handle sudden competitive situations [9].

Although long-term commitment to a single specialization can lead to advantages in skill and high-level performance, such as specific techniques and race adaptation, it also increases the risk of overuse injuries and psychological burnout [1,10,11]. In contrast, diversified training promotes the overall development of physical fitness through multidimensional stimuli, which may help individuals maintain a higher level of enjoyment and participation in sports [12]. Some studies suggest that early exposure to diversified training leads to more sustained competitive potential, whereas early specialization may lead to performance plateaus in later stages of development [13]. Although early specialization is essential in highly technical sports, such as gymnastics and figure skating, comprehensive physical development and skill transfer may be more beneficial for the long-term adaptability of young athletes [14,15].

In conclusion, the specialized training orientation represented by the SIE and the comprehensive physical fitness development emphasized by the PECEE correspond to two typical training pathways. The participants in this study were student-athletes who entered university through either the SIE or the PECEE and chose track-and-field sprinting as their specialization upon enrollment. While the participants differ in their admission routes and early training experiences, they share a common interest in sprinting and similar competitive goals. This unique characteristic provides an ideal condition for exploring the effects of different training pathways on sprinting performance.

Therefore, this study aimed to quantify and compare multidimensional biomechanical indicators between participants from two admission pathways to (1) delineate pathway-related differences in physical structure and performance mechanisms, and (2) identify key performance-limiting domains in the PECEE group that may serve as priorities for subsequent training and performance enhancement. Specifically, we addressed the following research questions: Do SIE and PECEE participants differ in reactive strength, neuromuscular activation characteristics, and postural control under standardized testing conditions, and if so, which domains most strongly distinguish the two groups beyond maximal strength capacity?

We hypothesized that participants in the SIE group would demonstrate superior reactive strength (e.g., higher RSI), faster neuromuscular activation or more efficient activation patterns, and better postural control than the PECEE group, despite comparable maximal strength, indexed by isokinetic peak torque. Accordingly, we further expected that any observed deficits in the PECEE group would highlight trainable targets that could be emphasized in their subsequent training to narrow the performance gap.

## 2. Materials and Methods

### 2.1. Participants

This cross-sectional study recruited 20 male participants aged 19 to 24 years from the School of Physical Education at Beijing Normal University. All participants selected track and field upon entering university and were assigned to the sprint discipline at matriculation. At the time of testing, all included participants had completed at least one full semester of supervised sprint training through the university track-and-field team training program. In addition, prior to university entry, all participants had received a minimum of three years of formal athletic training in their respective pre-university training pathways. Eligible participants were informed about the study procedures and provided written informed consent prior to screening and testing.

The inclusion criteria were (1) admission via either the Physical Education College Entrance Examination (PECEE) pathway or the Sports Independent Enrollment (SIE) pathway; (2) selection of sprinting as the primary specialization within the first year of enrollment and participation in regular systematic training under the university program (≥5 sprint/strength-conditioning sessions per week for ≥6 months); (3) a minimum competitive performance level, defined as a 100 m personal best of ≤11.30 s (within the past 12 months, obtained from official competition records or a standardized timed trial); and (4) stable health status allowing completion of all assessments. The exclusion criteria were (1) any lower-limb musculoskeletal injury within the past 6 months associated with pain, functional limitation, or requiring medical treatment, or injury-related cessation of training/substantial reduction in training volume within the previous 4 weeks; (2) any serious neurological or cardiovascular condition that could compromise testing safety; and (3) pain or discomfort on the testing day that prevented safe completion of the isokinetic assessment, drop jump protocol, or static balance tests.

Participants were categorized into the PECEE group or the SIE group according to their admission pathway. To ensure objective and verifiable grouping, the SIE group comprised participants admitted through the SIE pathway, who typically had earlier specialization and prior experience representing their school or provincial teams in provincial-level or national youth track-and-field competitions in China; the PECEE group comprised participants admitted through the PECEE pathway, whose pre-university training and assessment background is generally more diverse and may involve sprinting as well as other track-and-field or fitness-related components. These grouping criteria reflect commonly implemented selection features of the two admission pathways in Chinese university physical education programs; thus, within the context of our institution, the present sample may be considered broadly typical of male sprinters entering through these two routes.

It should be noted that this study was conducted at a single university and included only male participants who selected sprinting; therefore, generalization to female participants, other events, or other institutions should be made with caution.

This study was designed as a real-world cross-sectional comparison of sprinters entering the same university training environment via two distinct admission pathways. Because these pathways inherently differ in pre-university selection standards and training exposure, our primary aim was to characterize between-group functional differences and their potential associations with training exposure and anthropometrics, rather than to make strict causal inferences.

The participants’ basic physical measurements and baseline conditions are presented in Table 1. This study was approved by the Medical Ethics Committee of Wuhan Sports University (NO. 2025106) and was conducted in strict accordance with the ethical principles outlined in the Declaration of Helsinki.

### 2.2. A Priori Sample Size Estimation and Justification

This study conducted a sample size estimation using G*Power software (version 3.1.9.7). Based on prior research on drop jump and reactive strength outcomes [16], we anticipated a moderate between-group effect and therefore set the expected effect size to Cohen’s d = 0.61 for the a priori power analysis as a conservative assumption for the primary outcomes. We anticipated that the study may exhibit a medium to large effect size, and therefore set Cohen’s d to 0.61. The significance level was set at 0.05, and the statistical power was set at 0.95. Based on these parameters, the calculation indicated that a total sample size of 20 participants (10 per group) would be required. Due to the strict inclusion criteria—requiring participants to (1) be admitted through either the SIE or PECEE pathway, (2) specialize in track-and-field sprinting, and (3) possess no recent injuries—the available population within the university was limited. Thus, the sample size of 20 represents the maximum feasible number of eligible participants. This size also meets the a priori power requirement, ensuring sufficient statistical validity, but may be underpowered for small effects.

### 2.3. Study Design and Procedures

This study employed a cross-sectional, controlled design. All participants underwent testing on the same designated rest day, with a warm-up session required prior to testing to minimize the risk of injury. The testing procedure followed a standardized sequence, including isokinetic knee strength (quadriceps and hamstrings), a countermovement jump, and a 30 s static stance balance test. A 20 min rest interval was provided between each test to allow for adequate recovery. Participants were tested while wearing their own athletic shoes to maintain ecological validity.

### 2.4. Isokinetic Resistance Test

Isokinetic knee strength testing was performed using the isokinetic dynamometer (Biodex System 4, Biodex Medical Systems, Shirley, New York, NY, USA). The system has been widely used in strength and functional testing. According to the relevant literature, this device is capable of accurately measuring peak torque during knee flexion–extension and detecting muscle imbalances [17,18]. The test parameters were set to an angular velocity of 60°/s [19] to evaluate the participants’ maximal strength. Participants were instructed to perform five consecutive knee flexion–extension movements with maximal effort. During testing, participants were required to maintain stable knee flexion–extension motions to ensure the reliability and consistency of the results. The following parameters were recorded to assess muscle strength: the peak torque of the knee extensor, the peak torque of the knee flexor, the time to peak torque, and the hamstring/quadriceps ratio. It is recommended to add that the Biodex System 4 Pro, which was calibrated before each testing session in accordance with the manufacturer’s instructions.

### 2.5. Drop Jump Test

The drop jump test was conducted using Kistler Portable 3D force plates (Kistler Instrumente AG, Winterthur, Switzerland) and a Delsys Trigno™ Wireless EMG system (Delsys, Inc., Natick, MA, USA). All participants were familiar with the drop jump technique; they performed vertical jumps from three different platform heights: 20 cm, 40 cm, and 60 cm [20,21]. Immediately upon landing, participants performed a maximal vertical jump. Each height was tested three times, and the best value was selected for analysis; specifically, the trial with the highest reactive strength index (RSI) was used as the best value. During the test, participants were instructed to land with both feet parallel and to minimize knee flexion upon landing to ensure test reliability. The force platform recorded the ground reaction forces during landing, and the following key parameters were calculated: the RSI, ground contact time (GCT, s), flight time (FT, s), jump height (cm), take-off velocity (m·s^−1^), and leg stiffness index (kN·m^−1^), which were used to assess lower limb stiffness. Additionally, wireless sEMG electrodes were placed on the bellies of 16 muscles (Rectus femoris, Vastus lateralis, Vastus medialis, Semitendinosus, Tibialis anterior, Peroneus longus, Lateral gastrocnemius, Soleus), and muscle activation levels were recorded at three stages of the drop jump (landing, take-off, and flight). Surface EMG electrodes were placed according to the SENIAM guidelines. After shaving (if necessary) and cleaning the skin with alcohol, bipolar Ag/AgCl electrodes were positioned over the belly of each muscle, aligned with the presumed muscle fiber direction, with an inter-electrode distance of 20 mm. The mean and standard deviation of the muscle activation levels were analyzed to evaluate differences in force production patterns among participants.

### 2.6. Balance Ability Test

The balance ability test was conducted using Kistler Portable 3D force plates, and participants performed 30 s static standing trials in four different stances: double-leg eyes open, double-leg eyes closed, single-leg eyes open (dominant leg), and single-leg eyes closed (dominant leg). The takeoff leg used in the high jump and long jump events was defined as the dominant leg. Each stance was tested twice with a 1 min rest between trials. Participants were instructed to maintain a stable posture, avoiding unnecessary body sway or support movements during the test, and the last 10 s of stable data were extracted for analysis. If the participant placed both feet on the ground at any point within the 30 s, the trial was considered a failure and was repeated. The force platform recorded the center of pressure oscillation data, and the following key parameters were calculated: total excursion (TOTEX, mm^2^), mean distance (MDIST, mm), root mean square distance (RDIST, mm), and 95% confidence circle area (CC_AREA_95, mm^2^).

### 2.7. Data Analysis

#### 2.7.1. Drop Jump Test

The force platform data for the drop jump test were collected at a sampling frequency of 2000 Hz. The data processing procedure includes signal preprocessing, landing event detection, motion phase segmentation, and key parameter calculation. An improved multithreshold detection algorithm was used for signal preprocessing and landing event detection to identify drop jump landing events.

The data processing approach for the drop jump test was adapted from the study by Douglas et al. (2018) [22], with adjustments made based on the equipment available for this study. The following settings were applied: a minimum contact force threshold of 20 N, a significant landing threshold of 20 N, a minimum contact duration of 50 ms, and a minimum event separation time of 300 ms.

Lower limb stiffness was calculated using the classic spring-mass model [23], where *M* represents body weight (kg). After calculation, the data were normalized to eliminate the influence of body weight:

(1) $$K_{Leg}\;=\;\frac{M\cdot \pi \cdot \left(T_{flight}+T_{contact}\right)}{T_{contact}^{2}\cdot \left(\frac{T_{flight}+T_{contact}}{\pi }-\frac{T_{contact}}{4}\right)\cdot 1000}\;\left(\mathrm{in}\;kN\cdot m^{-1}\cdot {kg}^{-1}\right).$$

The electromyographic data processing for the drop jump involves removing the DC offset from the 16-channel surface electromyographic signals (sampling frequency: 2000 Hz), followed by a 30 Hz low-pass filter to extract the envelope. The signal is then normalized to the percentage of the maximal voluntary contraction.

Phase division of the depth jump was carried out as follows: the preactivation phase (150 ms before contact), the absorption phase (from initial contact to the peak of the vertical ground reaction force), and the propulsion phase (from the force peak to take-off). The actual take-off moment was verified using a 50 ms window threshold of 25 N to ensure the accuracy and reproducibility of phase division. The 16-channel surface electromyographic signals (sampling frequency: 2000 Hz) were processed using standardized digital signal processing. The processing steps for the raw EMG signals are summarized as follows: first, the DC offset was removed to eliminate baseline drift; a fourth-order Butterworth bandpass filter (20–450 Hz) was applied to remove motion artifacts and high-frequency noise; a second-order Butterworth notch filter (48–52 Hz) was applied to eliminate power-line interference; the filtered signals were rectified using a full-wave rectification function; a fourth-order Butterworth low-pass filter (30 Hz) was used to extract the EMG envelope; and the signal was normalized using the peak values of each channel, standardizing the activation level to the percentage of maximal voluntary contraction (%MVC). Participants performed MVCs in standardized postures appropriate for each target muscle while manual resistance opposed maximal isometric efforts. Contractions lasted 3–5 s with 2 min rest, repeated 2 times; the maximum RMS from filtered, rectified sEMG was taken as MVC. Task EMG was normalized to MVC and ×100 to obtain %MVC. The final normalized EMG envelope data were used for subsequent phase activation analysis. All the EMG data were processed using R (version 4.3.0) and the signal package, ensuring standardized data processing and reproducible results.

For each muscle and phase separately, EMG time series were compared between the dominant and nondominant sides using a two-sample t-test implemented in the open-source Python (version 3.11.9) package spm1d. The data were organized as three-dimensional arrays (subjects × muscles × normalized time points). SPM{t} curves were calculated, and statistical inference was performed at a significance level of α = 0.1 (two-tailed) using random field theory to determine the critical threshold. Clusters of the SPM{t} curve exceeding this threshold were considered significant, and their corresponding *p*-values were extracted. All results were visualized as SPM{t} plots with the threshold and cluster *p*-values indicated.

#### 2.7.2. Balance Test

For the balance test data, the raw force platform data (2000 Hz) were downsampled to 100 Hz. The multiscale entropy calculation method used by Lee et al. (2018) was referenced [24].

### 2.8. Statistical Analysis

Statistical analysis was performed using R (version 4.3) with the tidyverse and boot packages. Data were imported from a CSV file, and all numeric variables except the grouping variable and participant identifier were included in the analysis. The normality of each variable within each group was assessed using the Shapiro–Wilk test; variables with a *p*-value > 0.05 in both groups were considered normally distributed. And Spearman’s rank correlation coefficient was used to assess the relationships between drop jump variables and isokinetic strength variables.

For variables meeting the normality assumption, an independent-samples Welch’s t-test (unequal variances assumed) was used to compare the two groups. The effect size was quantified as Hedges’ g, and its 95% confidence interval was estimated via percentile bootstrap with 5000 resamples. For variables violating normality in at least one group, the Mann–Whitney U test was applied, and the effect size was expressed as the rank-biserial correlation (r_rb), also with bootstrap-based 95% confidence intervals.

Descriptive statistics are presented as the mean ± standard deviation for each group. To account for multiple comparisons, the Benjamini–Hochberg false discovery rate (FDR) correction was applied to the obtained *p*-values; adjusted q-values are reported. The complete results, including group sample sizes, means, standard deviations, test statistics, *p*-values, effect sizes, and their confidence intervals, were exported to a CSV file. Raw data are available from the corresponding author upon reasonable request. The effect size was calculated using G*power software (version 3.1.9.7; Heinrich Heine University, Düsseldorf, Germany).

## 3. Results

### 3.1. Isokinetic Strength Test

In the isokinetic strength test, a significant difference was observed between the SIE and PECEE groups in terms of the time to reach peak torque of the extensor on the nondominant side (*p* = 0.002, q = 0.028) (Table 2). No significant differences were found in the other parameters.

### 3.2. Drop Jump Test

In the drop jump test, Table 3 summarizes the key outcomes, including the reactive strength index (RSI), ground contact time (GCT), leg stiffness normalized to body weight, and propulsion duration; the complete set of variables is provided in the Supplementary Material. Across all drop heights (20, 40, and 60 cm), the SIE group demonstrated consistently higher RSI than the PECEE group. In parallel, the SIE group showed shorter GCT and shorter propulsion duration at each height, while leg stiffness normalized to body weight was greater across heights. These between-group differences remained significant after false discovery rate correction (q_FDR < 0.05). Notably, impact force/vertical ground reaction force outcomes were not included in the present analysis.

**Figure 1:**
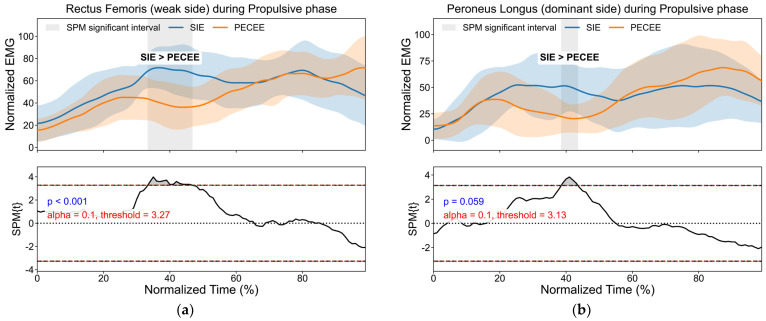

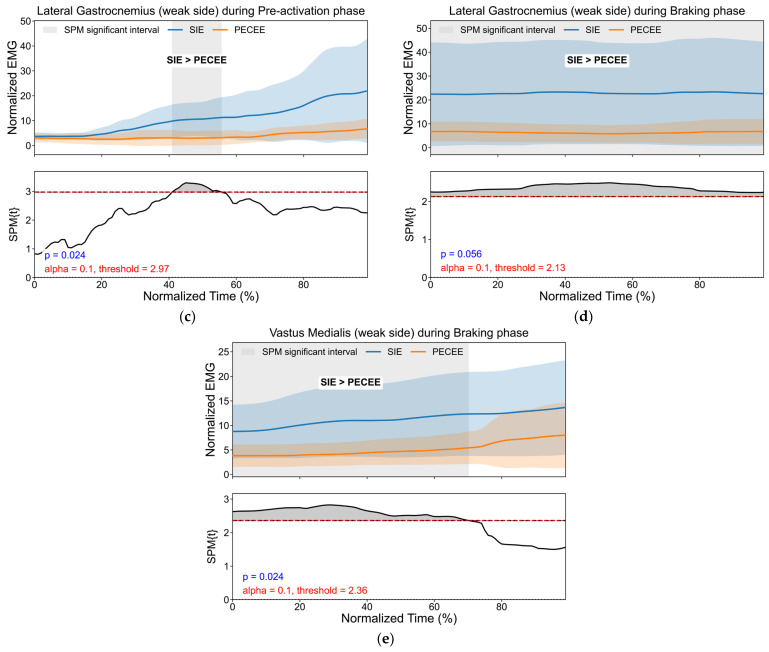
SPM analysis and EMG waveform of muscle activation levels during the drop jump. (**a**) The left rectus femoris during the propulsive phase; (**b**) The right peroneus longus in the propulsive phase; (**c**) The left lateral gastrocnemius during the preactivation phase; (**d**) The left lateral gastrocnemius during the braking phase; (**e**) The left vastus medialis during the braking phase. Note: The red dashed line indicates the statistical significance threshold, and the shaded regions denote time intervals showing statistically significant differences.

Regarding EMG activity across different phases of the drop jump, SPM analysis identified significant intergroup differences in several muscles, with muscle activation levels being greater in the SIE group than in the PECEE group (Figure 1). Specifically, the left rectus femoris significantly differed during the propulsive phase (Figure 1a, *p* < 0.001), with a significant interval spanning approximately 35–55% of the relative time. The right peroneus longus exhibited a near-significant difference in the propulsive phase (Figure 1b, *p* = 0.059). Moreover, the left lateral gastrocnemius significantly differed during the preactivation phase (Figure 1c, *p* = 0.024) and nearly significantly differed during the braking phase (Figure 1d, *p* = 0.056). Additionally, the left vastus medialis significantly differed during the braking phase (Figure 1e, *p* = 0.024), with a relatively long-lasting interval of significance.

**Figure 2:**
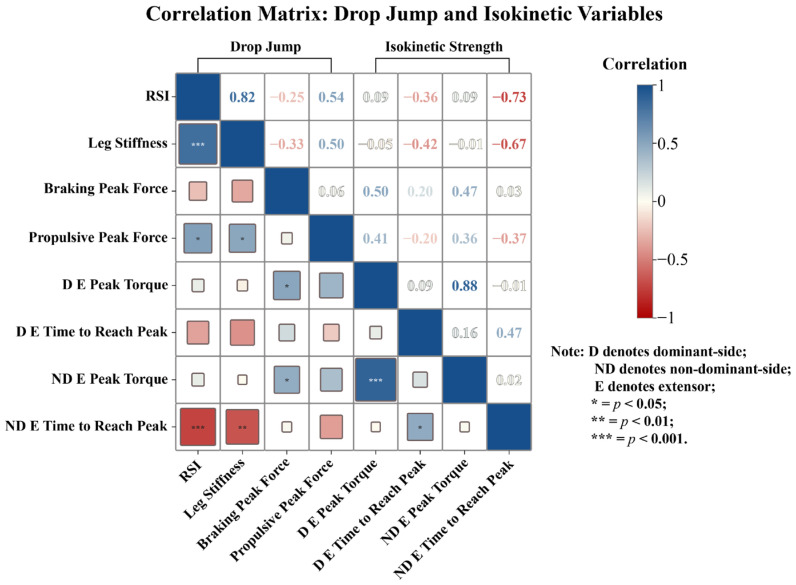
Correlation matrix: drop jump and isokinetic variables.

The correlations between the depth jump test metrics and isokinetic strength metrics are shown in Figure 2. The RSI was significantly negatively correlated with the time to peak torque of both the dominant and nondominant extensors (r = −0.58; *p* < 0.01). Moreover, leg stiffness was moderately negatively correlated with flexor torque–time parameters, whereas propulsive peak force was moderately positively correlated with extensor peak torque values (r = 0.44–0.50; *p* < 0.05). Braking peak force was positively correlated with peak extensor torque on both the dominant side (r = 0.50, *p* < 0.05) and the nondominant side (r = 0.47, *p* < 0.05).

### 3.3. Balance Test

In the balance test, Table 4 summarizes the key sway-related outcomes, including total excursion (TOTEX), mean distance (MDIST), radial distance (RDIST), and the 95% confidence circle area (CC AREA 95) across the four conditions. Under the most challenging condition (SLS-EC), the SIE group exhibited consistently lower TOTEX, MDIST, RDIST, and CC AREA 95 than the PECEE group, indicating reduced postural sway. In contrast, under SLS-EO and both double-leg stance conditions (DLS-EC and DLS-EO), no clear between-group differences were observed for these variables. After false discovery rate correction, however, none of the balance outcomes remained statistically significant (q_FDR > 0.05).

## 4. Discussion

### 4.1. Summary

This study sought to characterize and compare the multidimensional biomechanical profiles of participants entering through two admission pathways, with the broader aim of clarifying pathway-related differences in physical structure and performance strategies, while also identifying the principal performance constraints in the PECEE group that may warrant priority in subsequent training and performance enhancement. Overall, this objective was achieved. Consistent with our hypothesis, between-group differences were more evident in drop jump outcomes and phase-specific electromyography activation patterns than in the magnitude of isokinetic knee strength. Specifically, isokinetic knee strength indices were largely comparable between groups, whereas the Sports Independent Enrollment group showed a shorter time to reach peak torque in the nondominant knee extensors (Table 2). In the drop jump tests, the Sports Independent Enrollment group demonstrated higher reactive strength, shorter ground contact time, greater leg stiffness normalized to body weight, and shorter propulsion duration across heights (Table 3). Moreover, waveform-level electromyography analysis indicated phase-dependent differences in muscle activation patterns during preactivation, braking, and propulsion (Figure 1). Regarding postural control, group differences were primarily observed under the most challenging single-leg eyes-closed condition in unadjusted comparisons, but these did not remain significant after false discovery rate adjustment (Table 4).

Taken together, the present cross-sectional comparison suggests that pathway-associated advantages are expressed mainly in rapid force production and neuromuscular control rather than in isokinetic strength magnitude. From an applied perspective, these findings support a training focus for athletes entering via the Physical Education College Entrance Examination pathway that prioritizes improving rapid force generation capacity, refining activation timing and coordination during stretch–shortening actions, and enhancing sensorimotor control under challenging balance conditions.

Our findings explain that the participants with a specialized training background demonstrated advantages in performance-related domains that go beyond maximal strength, including reactive strength, neuromuscular activation characteristics, and postural control. These findings are consistent with the framework proposed by Berthelot et al. (2015), who suggested that long-term specialized training drives participants’ morphological and functional profiles toward event-specific optima, thereby enhancing performance determinants that are highly specific to the demands of each discipline [25]. In this view, specialization should not be interpreted as merely developing a narrow, sport-specific ability, but rather as a structured training process that integrates general physical conditioning with repeated sport-specific skill practice to build the coordinated set of qualities required for performance. Therefore, specialized training should not only enhance basic physical fitness (e.g., strength and endurance) but also integrate general physical training with specialized skill training, emphasizing coordination and reaction speed to improve overall performance [26]. This principle is also relevant to sprint-oriented disciplines, where performance depends on the effective expression of strength and power within highly specific coordinative patterns.

### 4.2. Isokinetic Strength Test

According to the isokinetic strength test results, both groups of participants exhibited a high degree of similarity. This suggests that despite differences in training background and modalities, comparable physiological adaptations in fundamental strength capacity were observed. Similar findings have been reported in studies involving participants from different sport disciplines. For example, no significant differences in knee isokinetic strength were found among elite female soccer, basketball, and handball players [27]. Similarly, although judokas and cyclists demonstrate distinct adaptations in trunk isokinetic strength and aerobic metabolism, their baseline levels remain comparable [28]. These findings support the findings of the present study, indicating that participants with different training backgrounds exhibit similar basic strength levels. Even when training methods vary substantially, no absolute advantage emerges in baseline physical fitness; instead, the primary differences are reflected in neuromuscular adaptations directly related to sport-specific performance.

### 4.3. Drop Jump Test

The drop jump results demonstrated that the SIE group consistently outperformed the PECEE group across all heights, exhibiting significantly higher RSI and shorter ground contact times (CTs), indicating superior stretch–shortening cycle (SSC) efficiency. With increasing drop height, between-group differences diminished, suggesting that the advantages of specialized training are primarily expressed under conditions requiring rapid force generation rather than maximal strength per se. These findings are consistent with the isokinetic test results, where no significant differences were observed in peak torque values between groups, but clear disparities emerged in torque–time parameters reflecting force production speed.

The correlation analysis shown in Figure 2 further supports this explanation, indicating that shorter contact times are closely associated with faster torque development, suggesting that greater reactivity is linked to quicker torque production. These findings suggest that explosive performance is more dependent on rapid neuromuscular activation and efficient energy utilization than on absolute strength capacity.

In addition, the SIE group demonstrated higher leg stiffness, which is closely related to improved force transmission and reduced energy loss during rapid movements. Although no significant group differences were observed in terms of flight time, jump height, or take-off velocity, compared with the PECEE group, the SIE group exhibited significantly shorter ground contact and propulsion times. These results suggest that SIE participants can achieve equivalent energy conversion within a shorter time frame, a characteristic that translates to sprint performance by enabling faster acceleration, reduced energy cost, and superior running economy. Previous studies support this perspective: Yamauchi et al. (2010) reported a strong association between the RSI and mean running velocity in endurance participants, emphasizing that greater reactive strength enhances elastic energy storage and release efficiency during SSC, thereby reducing metabolic energy expenditure (e.g., lactate accumulation) and improving running efficiency [29]. Similarly, in sprinting, greater vertical and leg stiffness allows participants to store ground reaction force more effectively as elastic potential energy. Stiffer lower limbs reduce excessive joint flexion and deformation, improving the recoil efficiency of elastic energy. This enables participants to rapidly convert stored elastic energy into propulsion, shorten ground contact time, and increase running speed [29,30]. In this study, statistical parametric mapping (SPM) was employed to analyze intergroup differences in muscle activation patterns and timing across different phases of the drop jump task. Unlike conventional mean-value analyses, SPM enables a refined time series examination, thereby offering a more precise understanding of the relationship between muscle activation and performance outcomes. The results revealed that the SIE group exhibited greater muscle activation across several key phases. In particular, the significantly increased activation of the rectus femoris on the nondominant side during the propulsion phase suggests enhanced knee extension velocity and lower-limb force production, ultimately contributing to increased leg stiffness. These findings align with those of previous studies indicating that efficient quadricep activation plays a decisive role in improving jumping performance in explosive tasks [31]. Moreover, Alnahdi et al. (2016) demonstrated that quadricep strength symmetry is closely related to the symmetry of the knee extension torque and vertical ground reaction force, further supporting the notion that differences in interlimb strength exacerbate asymmetrical loading patterns, which in turn influence performance [32]. Temporal analyses further demonstrated that the SIE group initiated activation of the nondominant rectus femoris and vastus medialis earlier and maintained activation longer during the propulsion phase. This pattern of preactivation and prolonged activation likely enhances neuromuscular control, enabling more rapid energy conversion immediately after ground contact, thereby reducing the contact time and increasing the explosive force output. Additionally, during the braking phase, greater activation of the nondominant gastrocnemius lateralis was observed in the SIE group, reflecting enhanced stability control for impact absorption. These results indicate a compensatory activation strategy in the SIE group, wherein increased neural drive to the nondominant limb muscles serves to optimize overall movement performance. Such adaptations may provide additional support for force generation and stability, thereby contributing to the superior dynamic performance observed in the SIE group.

### 4.4. Balance Test

The balance test results indicated that the SIE group demonstrated superior postural stability only under the single-leg, eyes-closed condition (*p* = 0.041), whereas no significant group differences were observed in the single-leg, eyes-open or double-leg stance tasks. This suggests that the benefits of specialized training emerge primarily when sensory integration faces its greatest challenge. These findings are consistent with those of Wang et al. (2022), who reported that visual feedback plays a more pronounced role under unstable conditions [33]. Specifically, during the single-leg eyes-closed task, the SIE group exhibited significantly greater stability across major parameters. In contrast, in the double-leg eyes-open condition, compared with the PECEE group, the SIE group had higher TOTEX values (1045.73 ± 178.3 vs. 932.26 ± 82.29) but lower MDIST values (3.91 ± 1.16 vs. 4.50 ± 1.56). This finding indicates that although the center of pressure (COP) path length was longer, the SIE group maintained a more stable distribution around the mean position During the single-leg eyes-closed stance, participants in the SIE group appeared to engage in more frequent muscle activation to maintain stability, whereas those in the PECEE group exhibited larger but slower COP displacements, suggesting reduced adjustment efficiency and a greater likelihood of imbalance. Under the double-leg stance, the SIE group displayed more frequent COP fluctuations but stronger suppression in the anterior–posterior direction, implying an active control strategy characterized by rapid corrective actions to prevent instability. These findings align with prior evidence. Bardal et al. (2016) demonstrated that proprioceptive accuracy was positively associated with micromovement variance of the upper limbs in healthy individuals, thereby enhancing motor control efficiency [34]. Similarly, Glofcheskie et al. (2017) suggested that individuals with superior postural control can rapidly recruit core muscles to cope with sudden load perturbations, which helps maintain stability during high-intensity tasks and minimizes energy waste [35]. Taken together, our results indicate that the PECEE group relies more heavily on visual feedback, whereas the SIE group demonstrates enhanced proprioceptive ability. Such an advantage is likely transferable to dynamic locomotor tasks: during running or sprinting, the SIE group may better sense and regulate body posture and adapt to changes in speed, stride length, and foot strike, thereby improving forward propulsion efficiency while minimizing lateral energy loss, ultimately optimizing performance outcomes.

### 4.5. Significance

The present findings have important implications for athlete development and training program design. For students who enter university through physical education entrance examinations, targeted interventions aimed at improving reactive strength may help narrow the performance gap with their peers who possess a more specialized training background. In particular, plyometric training programs that emphasize short ground contact times and high-intensity stretch–shortening cycles (SSCs) may be especially effective. Moreover, the similarity observed in strength and aerobic capacity between the two groups suggests that students from a multisport training background already possess the fundamental physical capacities required for further specialization. These results support the notion that late specialization, while potentially disadvantageous in terms of immediate performance, does not preclude the attainment of elite-level capabilities when appropriate training interventions are applied.

### 4.6. Limitations

Several limitations should be acknowledged. First, the cross-sectional design restricts causal inference regarding the relationship between training pathways and performance-related outcomes. Accordingly, the observed between-group differences should not be interpreted as definitive training effects, because they may partly reflect selection bias and pre-existing characteristics associated with the admission pathways rather than the impact of subsequent university training. Our results therefore describe associations based on observations at a single time point; prospective longitudinal studies are required to determine how pathway-related differences emerge and evolve over time and to clarify the long-term impact of different training modalities on athletic performance.

Second, the relatively small sample size limits statistical power, particularly for detecting interaction effects and subtle between-condition differences, and increases the risk of Type II error. It also constrains subgroup analyses and reduces generalizability. In addition, participants were recruited from a single top-tier university in China and were selected based on outstanding achievements within two distinct admission pathways. In this sense, their performance may represent the upper-end outcomes of these two training models; however, the findings may not generalize to students who did not achieve top admission results, participants from other institutions or competitive levels, individuals with mixed or non-standard training histories, or participants trained under different national systems and sociocultural contexts. More broadly, this study could not fully capture differences across diverse backgrounds, characteristics, and contexts.

Third, training background was assessed retrospectively and operationalized primarily through the admission pathway as a proxy for prior training exposure. Detailed information such as training volume, intensity, periodization, sport-specific content, and injury exposure was unavailable, which may have introduced exposure misclassification. Fourth, only male participants were included; therefore, the findings should not be extrapolated to female athletes given known sex-related differences in neuromuscular control and injury risk profiles. Fifth, our assessment emphasized lower-limb function and postural-control outcomes and did not comprehensively evaluate sport-specific technical skills or upper-body capabilities, which may also contribute meaningfully to performance. Finally, several potential confounders were not directly measured or controlled, including genetic predisposition, coaching quality, motivation and other psychosocial variables, sleep, and additional lifestyle factors that may differ between groups and influence balance and neuromuscular outcomes. Future research should adopt prospective longitudinal or randomized designs, recruit larger multi-center cohorts including both sexes, collect detailed training logs, and incorporate relevant biological, injury-related, and psychosocial covariates to better isolate training-related effects and reflect the multifactorial nature of athletic development.

## 5. Conclusions

Although participants from diverse training backgrounds and with different performance goals exhibit comparable levels of maximal strength, sport-specific training confers distinct advantages in terms of reactive strength and complex postural control. Importantly, these performance gaps can be effectively mitigated through targeted neuromuscular training interventions. For sprint athletes who undergo late specialization, we recommend the systematic implementation of reactive strength-oriented training strategies to fully unlock their performance potential. From a long-term developmental perspective, diversified early training experiences contribute to a more comprehensive athletic foundation; however, this approach should be followed by the scientifically timed and progressive integration of specialized training components. Therefore, coaches and physical educators should emphasize the appropriate timing and programming of sport-specific training while maintaining a base of comprehensive physical conditioning to optimize athletic development outcomes.

## Figures and Tables

**Table 1:** Descriptive information for participants (*n* = 10 per group).

Variable	SIE Group	PECEE Group	Effect Size [95% CI]
Age (y)	21.3 ± 1.89	19.7 ± 0.95	0.53 [0.08, 0.88]
Height (cm)	177 ± 5.14	182.3 ± 5.96	−0.91 [−1.9, −0.16]
Body mass (kg)	67.75 ± 6.9	73 ± 7.82	−0.68 [−1.93, 0.14]
Training duration (y)	7 ± 3.6	4 ± 1.1	0.58 [0.2, 0.88]
100 m time (s)	10.75 ± 0.18	11.13 ± 0.21	−1.91 [−3.72, −1.07]

**Table 2:** Isokinetic Strength Test Results.

Variable	SIE Group	PECEE Group	*p*	Effect [95% CI]	q_FDR
Standardized DS F Peak Torque (N·m·kg^−1^)	191.96 ± 30.121	197.81 ± 36.975	0.940	−0.02 [−0.54, 0.52]	0.956
DS F Time to Reach Peak Torque (ms)	385 ± 162.224	395 ± 140.574	0.885	−0.06 [−0.94, 0.88]	0.956
Standardized NDS F Peak Torque (N·m·kg^−1^)	179.13 ± 35.384	188.73 ± 40.14	0.705	−0.10 [−0.62, 0.44]	0.956
NDS F Time to Reach Peak Torque (ms)	354 ± 154.431	428 ± 212.488	0.565	−0.15 [−0.64, 0.38]	0.956
Standardized DS E Peak Torque (N·m·kg^−1^)	296.36 ± 34.95	297.97 ± 82.43	0.956	−0.02 [−0.98, 0.89]	0.956
DS E Time to Reach Peak Torque (ms)	330 ± 117.189	430 ± 169.312	0.144	−0.66 [−1.65, 0.14]	0.576
Standardized NDS E Peak Torque (N·m·kg^−1^)	290.66 ± 49.339	304.45 ± 52.785	0.554	−0.26 [−1.19, 0.62]	0.956
NDS E Time to Reach Peak Torque (ms)	336 ± 97.775	517 ± 126.759	0.002 *	−1.53 [−2.57, −0.89]	0.028 *
Left Side F/E Muscle Strength Ratio	0.647 ± 0.065	0.693 ± 0.146	0.382	−0.39 [−1.24, 0.55]	0.956
Right Side F/E Muscle Strength Ratio	0.626 ± 0.12	0.623 ± 0.102	0.762	0.08 [−0.44, 0.60]	0.956
F Strength Ratio	1.156 ± 0.081	1.133 ± 0.113	0.609	0.22 [−0.58, 1.31]	0.956
E Strength Ratio	1.101 ± 0.088	1.197 ± 0.174	0.139	−0.39 [−0.80, 0.14]	0.576

Note: DS denotes dominant side, NDS denotes nondominant side, F denotes flexion, and E denotes extension; * indicates significant differences between the groups, * = *p* < 0.05.

**Table 3:** Comparison of Differences between the Two Groups in the Drop Jump Test.

Variable	Height (cm)	SIE Group	PECEE Group	*p*	Effect [95% CI]	q_FDR
RSI	20	1.805 ± 0.552	1.322 ± 0.2	0.0241 *	1.114 [0.579, 2.025]	0.024 *
	40	1.816 ± 0.584	1.346 ± 0.243	0.0102 *	0.68 [0.28, 0.96]	0.014 *
	60	1.536 ± 0.485	1.186 ± 0.156	0.0233 *	0.6 [0.16, 0.94]	0.024 *
Ground Contact Time (s)	20	0.312 ± 0.083	0.436 ± 0.043	0.0009 ***	−1.801 [−3.035, −1.157]	0.006 **
	40	0.327 ± 0.083	0.443 ± 0.06	0.0024 **	−1.532 [−2.72, −0.833]	0.006 **
	60	0.394 ± 0.088	0.507 ± 0.061	0.004 **	−1.44 [−2.51, −0.802]	0.006 **
Leg Stiffness to body weight (kN·m^−1^·kg^−1^)	20	0.173 ± 0.096	0.08 ± 0.012	0.0132 *	1.308 [0.894, 2.161]	0.016 *
	40	0.158 ± 0.095	0.079 ± 0.02	0.0025 **	0.8 [0.48, 1]	0.006 **
	60	0.11 ± 0.066	0.062 ± 0.013	0.0041 **	0.76 [0.4, 1]	0.006 **
Propulsion Duration (s)	20	0.288 ± 0.078	0.403 ± 0.048	0.0025 **	−0.8 [−1, −0.44]	0.006 **
	40	0.307 ± 0.079	0.426 ± 0.06	0.0015 **	−1.618 [−2.864, −0.947]	0.006 **
	60	0.381 ± 0.087	0.497 ± 0.06	0.0034 **	−1.472 [−2.51, −0.857]	0.006 **

* indicates significant differences between the groups; * = *p* < 0.05; ** = *p* < 0.01; *** = *p* < 0.001.

**Table 4:** Analysis of differences in key balance test parameters.

Variable	Condition	SIE Group	PECEE Group	*p*	Effect [95% CI]	q_FDR
TOTEX (mm)	SLS-EC	1397.4 ± 782.4	2893.87 ± 2002.79	0.004 *	−0.76 [−1, −0.36]	0.065
	SLS-EO	1002.12 ± 241.24	980.21 ± 223.83	0.406	0.22 [−0.32, 0.74]	0.611
	DLS-EC	1104.68 ± 146.36	1067.83 ± 198.91	0.643	0.2 [−0.6, 1.37]	0.735
	DLS-EO	1045.73 ± 178.3	932.26 ± 82.29	0.091	0.78 [0.08, 1.63]	0.292
MDIST (mm)	SLS-EC	16.25 ± 8.56	26.27 ± 11.41	0.034 *	−0.56 [−1, −0.08]	0.137
	SLS-EO	11.06 ± 3.8	11.54 ± 2.25	0.737	−0.15 [−1.26, 0.66]	0.786
	DLS-EC	4.89 ± 2.48	5.92 ± 3.18	0.496	−0.18 [−0.68, 0.34]	0.611
	DLS-EO	3.91 ± 1.16	4.5 ± 1.56	0.350	−0.41 [−1.43, 0.45]	0.611
RDIST (mm)	SLS-EC	19.57 ± 11.09	33.35 ± 13.01	0.019 *	−0.62 [−1, −0.16]	0.102
	SLS-EO	13.03 ± 5.13	13.1 ± 2.59	0.968	−0.02 [−1.21, 0.79]	0.968
	DLS-EC	5.86 ± 2.94	7.39 ± 4.1	0.450	−0.2 [−0.68, 0.34]	0.611
	DLS-EO	4.68 ± 1.36	5.27 ± 1.79	0.422	−0.35 [−1.36, 0.53]	0.611
CC AREA 95 (mm^2^)	SLS-EC	4609.9 ± 7082.23	12,467.44 ± 10,406	0.010 *	−0.68 [−1, −0.28]	0.081
	SLS-EO	1800.51 ± 1624.8	1523.9 ± 631.55	0.496	−0.18 [−0.68, 0.38]	0.611
	DLS-EC	393.63 ± 454.73	692.59 ± 741.8	0.406	−0.22 [−0.72, 0.34]	0.611
	DLS-EO	221.58 ± 128.02	278.5 ± 172.54	0.414	−0.36 [−1.38, 0.5]	0.611

Note: SLS-EC denotes single-leg stance with eyes closed, SLS-EO denotes single-leg stance with eyes open, DLS-EC denotes double-leg stance with eyes closed, and DLS-EO denotes double-leg stance with eyes open; * indicates significant differences between the groups; * = *p* < 0.05.

## Data Availability

The data from this study are available from the corresponding author upon request, but are not publicly available due to privacy restrictions.

## Supplementary Materials

The following supporting information can be downloaded at https://www.mdpi.com/article/10.3390/s26061906/s1.

## Abbreviations

The following abbreviations are used in this manuscript:

PECEE	Physical Education College Entrance Examination
SIE	Sports Independent Enrollment
RSI	Reactive Strength Index
GCT	Ground Contact Time
FT	Flight Time
SSC	Stretch–Shortening Cycle
sEMG	Surface Electromyography
EMG	Electromyography
COP	Center of Pressure
TOTEX	Total Excursion
MDIST	Mean Distance
RDIST	Root Mean Square Distance
CC_AREA_95	95% Confidence Circle Area
DLS	Double-Leg Stance
SLS	Single-Leg Stance
EO	Eyes Open
EC	Eyes Closed
DS	Dominant Side
NDS	Nondominant Side
SPM	Statistical Parametric Mapping
CI	Confidence Interval
SD	Standard Deviation
